# Validation of Serum Biomarkers Derived from Proteomic Analysis for the Early Screening of Preeclampsia

**DOI:** 10.1155/2015/121848

**Published:** 2015-01-05

**Authors:** Aggeliki Kolialexi, Dimitrios Gourgiotis, George Daskalakis, Antonis Marmarinos, Alexandra Lykoudi, Danai Mavreli, Ariadni Mavrou, Nikolas Papantoniou

**Affiliations:** ^1^Department of Medical Genetics, Athens University, “Aghia Sofia” Children's Hospital, 11527 Athens, Greece; ^2^2nd Department of Pediatrics, Athens University, 11527 Athens, Greece; ^3^1st Department of Obstetrics & Gynecology, Athens University, 11528 Athens, Greece

## Abstract

*Aim.* To examine the potential value of previously identified biomarkers using proteomics in early screening for preeclampsia (PE). *Methods.* 24 blood samples from women who subsequently developed PE and 48 from uncomplicated pregnancies were obtained at 11–13 weeks and analysed after delivery. Cystatin-C, sVCAM-1, and Pappalysin-1 were quantified by ELISA. Maternal characteristics and medical history were recorded. *Results.* Median values of Cystatin-C, sVCAM-1, and Pappalysin-1 in the PE group as compared to controls were 909.1 gEq/mL versus 480.0 gEq/mL, *P* = .000, 832.0 gEq/mL versus 738.8 gEq/mL, *P* = .024, and 234.4 gEq/mL versus 74.9 gEq/mL, *P* = .064, respectively. Areas under the receiver-operating characteristic curves (AUC, standard error (SE)) for predicting PE were Cystatin-C: 0.90 (SE 0.04), VCAM-1: 0.66 (SE 0.074), and Pappalysin-1: 0.63 (SE 0.083). To discriminate between cases at risk for PE and normal controls, cut-off values of 546.8 gEq/mL for Cystatin-C, 1059.5 gEq/mL for sVCAM-1, and 220.8 gEq/mL for Pappalysin-1 were chosen, providing sensitivity of 91%, 41%, and 54% and specificity of 85%, 100%, and 95%, respectively. *Conclusions.* sVCAM-1 and Pappalysin-1 do not improve early screening for PE. Cystatin-C, however, seems to be associated with subsequent PE development, but larger studies are necessary to validate these findings.

## 1. Introduction

Preeclampsia (PE) is a pregnancy-specific syndrome characterized by hypertension and proteinuria, developing after the 20th week of gestation, in a previously normotensive woman. PE may have an early (<34 weeks of gestation) or a late (>34 weeks of gestation) onset. The condition complicates approximately 2–8% of pregnancies and is one of the major causes of maternal, fetal, and neonatal morbidity and mortality [1, 2]. Early identification of patients with an increased risk for PE is therefore one of the most important goals in obstetrics.

Multiple pathological pathways contribute to the final phenotype of PE. These include poor placental perfusion, defective remodelling of uteroplacental vessels in early pregnancy, maternal genetic predisposition, and vascular endothelial dysfunction [3].

Various biochemical markers have been proposed for the identification of women at risk for PE [4–10]. The majority were chosen on the basis of specific pathophysiological abnormalities that have been reported in association with PE, such as placental dysfunction, endothelial and coagulation activation, and systemic inflammation. The concentration of these biomarkers in maternal serum has been found either increased or decreased early in gestation before the onset of PE. Findings, however, have been inconsistent and none of the suggested markers have demonstrated sufficient sensitivity and specificity to allow identification of pregnancies that would benefit from increased monitoring or early preventive therapies.

Research in recent years has moved toward unbiased “systems medicine” approaches, using hypothesis-generating strategies to investigate new pathways. One promising technique is proteomics, the simultaneous analysis of thousands of peptides and proteins. Several biological fluids and fetal tissues have been successfully tested, including maternal plasma, urine, and CVS and several potential biomarkers for the early prediction of PE have been identified [11–13]. Kolla et al. observed elevation in 10 proteins in the PE group, as compared to normotensive control pregnant women [14]. These included clusterin, fibrinogen, fibronectin, angiotensinogen, and galectin 3, increased levels of which are known to be associated with PE. In another study, Blumenstein et al. used a combination of immunodepletion and 2D DIGE and identified 39 differentially expressed proteins in the plasma of women at 20 weeks of gestation who subsequently developed PE [15]. These proteins were mainly involved in lipid metabolism, coagulation, complement regulation, extracellular matrix remodelling, protease inhibitor activity, and acute-phase responses. Another prospective study by Rasanen et al., using serum proteomics, showed distinct maternal serum proteomic profiles associated with preclinical and/or clinical PE [16].

The aim of the present study was to evaluate three potential proteomic biomarkers, namely, Cystatin-C (P01034), vascular cell adhesion protein-1 (sVCAM-1) (P19320), and Pappalysin-1 (Q13219). These proteins were chosen from a list of potential biomarkers for the detection of women with early and severe preclinical and/or clinical PE provided by Rasanen et al., for which monoclonal antibodies were commercially available. Cystatin-C and sVCAM-1 were found to be substantially increased in maternal serum in PE cases while levels of Pappalysin-A were decreased.

## 2. Material and Methods

In this retrospective and case-control study, peripheral blood samples were obtained from 1050 Caucasian pregnant women, during their first routine hospital visit at 11–13 weeks of gestation (mean 12.1 ± 0.6 weeks) in a large maternity hospital. Following a 5 min centrifugation at 2000 rpm, the cleared maternal serum was collected, aliquoted, and stored at 80°C.

Clinical data on these patients were collected by the obstetricians involved in the project, using a preestablished questionnaire [17]. All participants were normotensive at the time of blood sampling and had no renal dysfunction or proteinuria.

When pregnancy outcome was known from hospital medical records, it was revealed that 24 out of 1050 women developed PE, 2 of which required delivery at <32 weeks of gestation. Cystatin-C, sVCAM-1, and Pappalysin-A were measured in the 24 samples coming from these women and from 48 unaffected controls (1 : 2 ratio). Samples used as controls came from women with similar gestational age and duration of storage as those of the study group. All samples were analysed in duplicate. None of the samples were previously thawed and refrozen.

PE was defined according to the World Health Organization guidelines, which include an elevated systolic (≥140 mmHg) and/or diastolic (≥90 mmHg) blood pressures on repeated measurements and proteinuria (≥2+ as measured by dipstick).

The study was approved by the Athens University Committee on Ethics and in all cases written informed consent was obtained prior to blood drawing.

## 3. Methods

### 3.1. Enzyme-Linked Immunoassays

Cystatin-C, VCAM-1, and Pappalysin-A were quantified using the respective Human Cystatin-C, Human sVCAM-1, and Human Pappalysin-A assays (R&D Systems, Abington, UK) according to the manufacturer's protocols. Following a 15 min centrifugation at 16 000 g, the cleared serum was tested in duplicate at a dilution of 1 : 1 for Cystatin-C, 1 : 20 for sVCAM-1, and 1 : 30 for Pappalysin-A. Assay sensitivities were 0.102 ng/mL for Cystatin-C, 0.6 ng/mL for sVCAM-1, and 0.053 ng/mL for Pappalysin-A. The intra-assay coefficients of variation were 4.6% for Cystatin-C, 3.5% for sVCAM-1, and 4.3% for Pappalysin-A.

### 3.2. Statistical Analysis

Descriptive statistics for continuous parameters consisted of medians, whereas categorical variables were expressed as percentages. Patient characteristics of cases and controls were compared with the Mann-Whitney rank test for continuous variables or the Fisher's exact test for categorical variables, when appropriate. Comparisons of the biochemical data between the two study groups were performed with the Mann-Whitney rank test. Subsequently, receiver-operating characteristic (ROC) curves were calculated, and values for area under curve (AUC) with corresponding standard errors were estimated. Binary logistic regression was performed in order to determine the effect of each variable studied on risk assessment for PE. Statistical analysis was performed with the Statistical Package for Social Sciences version 20.0 (SPPS Inc., Chicago, IL, USA).

## 4. Results

**Figure 1:**
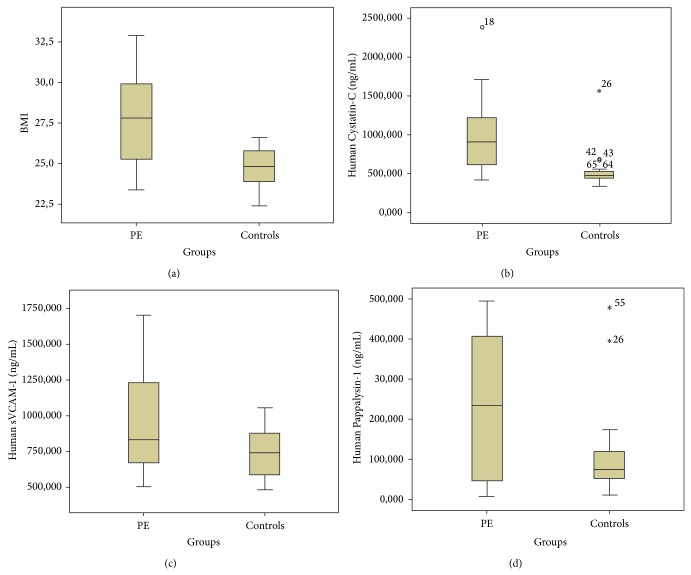
Box-plot of maternal prepregnancy BMI (a), Cystatin-C (b), VCAM-1 (c), and Pappalysin-A (d) in 24 pregnant women that subsequently developed PE and 48 uncomplicated pregnancies. The box represents the lower and upper quartiles, the medians are indicated by a line inside each box; the whiskers represent the 10th and 90th percentiles. Outliers, depicted as asterisks, are values more than 1.5 box lengths from either ridge of the box.

Maternal and neonatal characteristics of cases and controls included in the study are presented in Table 1. There was no difference in maternal age between patients who developed PE and controls, but prepregnancy body mass index (BMI) was significantly higher in the PE group (*P* < .001). Median BMI was 27.8 kg/m^2^ (range 9.5) in PE cases and 24.8 kg/m^2^ (range 4.2) in controls (Figure 1(a)). Furthermore, more women were nulliparous in the PE group as compared to controls. Thirty-five women in the control group and 12 in the PE group declared smoking regularly during pregnancy. As expected, patients with PE gave birth earlier than controls. Seven delivered preterm (<37 weeks of gestation) and two before the 34th week, at 27 and 30 weeks, respectively, due to uncontrollable high blood pressure and generalised edema. Both neonates, although premature, survived the first month after birth. Women in the control group delivered at term. The newborn's birth weight was lower in women with PE as compared to controls. Birth weight of 11 newborns from women who developed PE was at or below the 10th percentile. Additionally in 2 cases birth weigh was below the 5th percentile. All growth restricted neonates survived.

**Figure 2:**
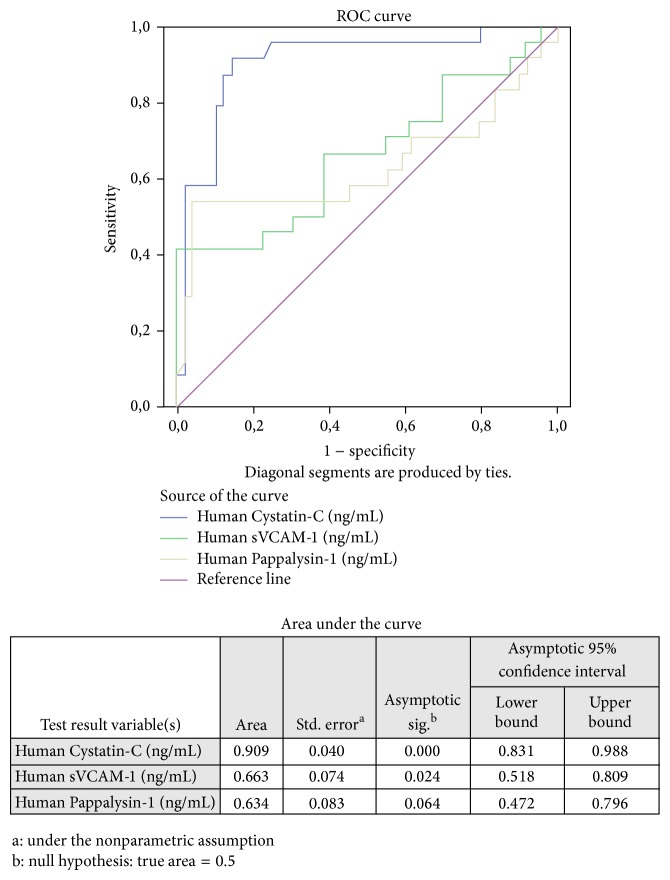
Receiver-operating characteristics (ROC) curves showing the sensitivity and specificity of first-trimester Cystatin-C, VCAM-1, and Pappalysin-A as biomarkers for the prediction of women at risk of PE.

Serum levels of Cystatin-C and VCAM-1 were significantly higher in patients with PE as compared to gestational age-matched controls (Figures 1(b) and 1(c)). The median concentration of serum Cystatin-C was 909.173 ng/mL (range 1964.1 ng/mL) in PE cases and 480.0 ng/mL (range 1231.610 ng/mL) in controls (*P* < .001). The median concentration of VCAM-1 was 832.3 ng/mL (range 1193 ng/mL) in PE cases and 738.8 ng/mL (range 563.7 ng/mL) in controls (*P* = .024). Maternal levels of Pappalysin-A did not differ significantly between PE cases (median 234.4 ng/mL, range 485.9 ng/mL) and controls (median 74.9 ng/mL, range 466.9 ng/mL) (*P* = .064) (Figure 1(d)). ROC curves were calculated and AUC values with corresponding standard errors are presented in Figure 2. In order to discriminate between cases at risk for PE and normal controls, cut-off values of 546.880 ng/mL for Cystatin-C, 1059.880 ng/mL for sVCAM, and 220.854 ng/mL for Pappalysin-A were chosen, which provided sensitivity of 91.7%, 41.7%, and 54.2% and specificity of 85.7%, 100%, and 95%, respectively.

Apart from the models analyzed using as input the measurements of each protein independently, 3 additional models were considered for binary regression analysis. The results obtained showed that all three new models had better performance than the simple random sampling procedure (Asymp. Sig < 0,05 in the above AUC). When binary logistic regression was performed using the patients' clinical data (BMI, smoking, and parity), risk assessment for PE was found to be statistically affected by maternal BMI. This model allowed the correct classification of 87.7% of women in the PE or the control group. Taking into consideration the data obtained from biomarker analysis (Cystatin-C, Pappalysin-A, and sVCAM-1) risk assessment for PE was statistically affected only by Cystatin-C. This model allowed the correct classification of 84.9% of pregnant women in the PE or the normotensive group. When all variables (biochemical and clinical) were taken into consideration, risk assessment for PE was found to be statistically affected by Cystatin-C and maternal BMI. The model which includes these parameters allows for the classification of 93.3% of pregnant women in the normotensive or the PE group.

When all the above models were analyzed after excluding the 2 cases with early PE, the variables that affect the probability of having PE were found to be similar in all three models.

**Figure 3:**
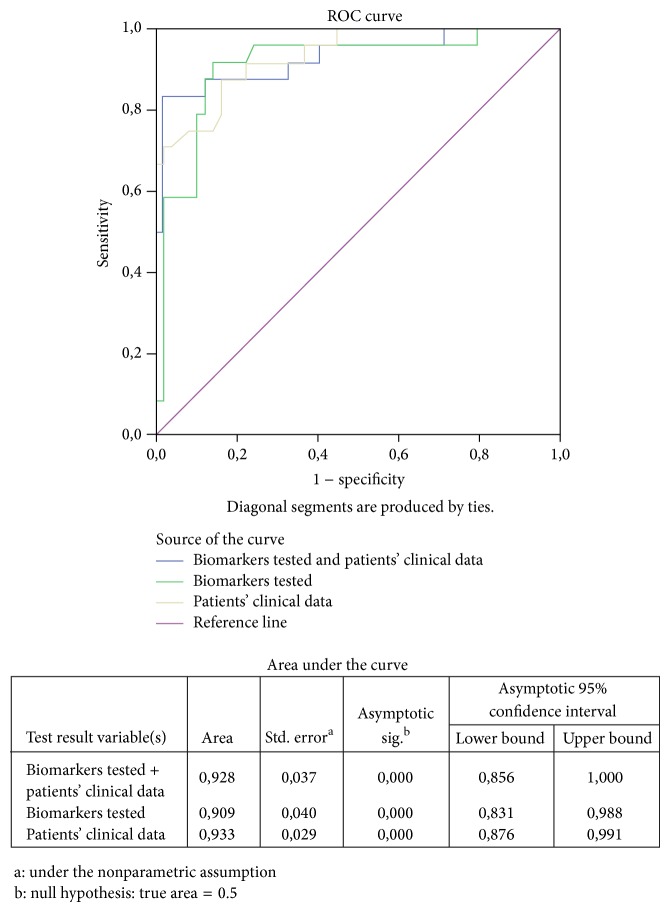
Receiver-operating characteristics (ROC) curves showing the sensitivity and specificity of first-trimester testing using A. patients' clinical characteristics, B. biochemical markers, and C. clinical and biochemical variables simultaneously, for the prediction of women at risk of PE.

All models showed similar AUC values implying that they may all estimate statistically accurately the risk assessment on PE (Figure 3). Since the model which includes patients' clinical data and biochemical measurements allows the correct classification of 93.3% of pregnant women in the PE or the normotensive group, it should be considered as the most preferable one.

## 5. Discussion

In the present retrospective study, 1st trimester serum levels of Cystatin-C, VCAM-1, and Pappalysin-A in women who subsequently developed PE and gestational age-matched controls were analysed. These proteins were chosen from the list of potential biomarkers for PE provided by Rasanen et al. for the identification of women with early and severe preclinical and/or clinical PE, since they are involved in different pathways leading to PE, showing statistically significant differential expression in PE cases, and monoclonal antibodies for their measurement are commercially available [16].

In the PE group, 22 out of 24 patients developed late-onset PE which is significantly more common and, despite being often mild, can be associated with significant clinical morbidity. Two patients manifested PE between 32 and 34 weeks of gestation but these two cases do not significantly affect the results of this study.

Our findings indicate that circulating VCAM-1 levels were significantly increased in PE as compared to normal pregnancy. Lyall et al. were the first to show that sVCAM-1 was elevated in the serum of patients with severe PE, as compared to the mild form of the disease, or normal pregnancy [18]. Krauss et al. found significantly elevated levels of sVCAM-1 in the plasma of pregnant women 3–15 weeks before the onset of clinical symptoms of PE [19]. sVCAM-1 is a member of the immunoglobulin superfamily and functions as a transmembrane receptor in vascular endothelial cell membranes. Increased concentrations of sVCAM-1 may reflect increased expression of this protein on the endothelial surface. In contrast, Haller et al. reported that sVCAM-1 expression was not increased in the serum of PE patients [20].

In the present study, the levels of Pappalysin-A in early pregnancy maternal serum in women that subsequently developed PE were decreased. However, since the association between Pappalysin-A serum levels and PE was relatively weak, the clinical usefulness of this marker is limited. Spencer et al. reported a small increase in likelihood ratio of developing PE with decreasing levels of Pappalysin-A [21]. More recent studies, however, have shown that although reduced first-trimester serum levels of Pappalysin-A are associated with PE, levels are also low in other complications of pregnancy [22–24]. It has been suggested that Pappalysin-A is more useful as a marker of IUGR than of PE.

Our data show that Cystatin-C could be a reliable first-trimester marker for PE. Cystatin-C is a protease inhibitor widely used by clinicians as a sensitive marker for renal function and for the estimation of glomerular filtration rate. Since all women had normal renal function, increased levels of Cystatin-C may be attributed to an increased placental production. This supports the hypothesis that the balance between trophoblast protease production and decidual protease inhibitor activity may have an important biologic role in trophoblast development and a derangement in this balance may predispose to poor trophoblast development and PE [25, 26].

Consistent with previous reports, a significant increased maternal BMI was found in women at risk for PE [27–29]. Statistical analysis showed that although the use of maternal clinical characteristics for risk assessment for PE showed higher AUC as compared to each of the 3 biochemical markers measured in the present study, simultaneous testing using maternal BMI and Cystatin-C can correctly classify 93.3% of pregnant women in the PE or the normotensive group.

The major limitations of the present study are the small number of samples analysed and the fact that clinical data known to be associated with PE, such as 1st trimester maternal mean arterial pressure (MAP) and mean uterine artery (UtA) Doppler pulsatility index (PI) measurements, are missing. Studies with larger sample sizes, especially with early onset PE, will be necessary to assess the definitive potential of these biomarkers for early detection of PE and risk assessment.

Since PE is a multifactorial and polysystemic pregnancy complication, more than one biomarker is needed in order to identify women at risk. As already mentioned, the 3 biomarkers tested in the present study were chosen on the basis of their statistically significant differential expression using proteomics. Nevertheless, other potential biomarkers, already identified using proteomics, should be validated.

## Figures and Tables

**Table 1:** Maternal and neonatal characteristics of cases and controls included in the study [17].

	Controls (*n* = 48)	PE (*n* = 24)	Statistics
Maternal age (y)	32.2 [28.8–37.3]	32.9 [27.3–35.0]	NS
Prepregnancy BMI (kg/m^2^)	24.8 [22.5–26.2]	27.9 [23.4–32.9]	*P* = .000
Cigarette smoker	35 (73%)	12 (50%)	*P* = .028
Nulliparous	29 (60.4%)	22 (91.6%)	*P* = .013
Parous	19 (39.6%)	2 (8.4%)	
Family history of PE	0	0	—
Conception			
Spontaneous	42 (87.5%)	24 (100%)	*P* = .090
IVF	6 (12.5%)	0	
Medical history			
Diabetes mellitus	0	0	—
Thrombophilia	0	1	
Mode of delivery			
Vaginal birth (*n*)	33 (68.7%)	8 (33.3%)	*P* = .013
Caesarean delivery (*n*)	15 (31.3%)	16 (66.6%)	
Gestational age at delivery (wk)	40.2 [37.2–42.0]	38.1 [32.1–40.6]	
<37 wk (*n*)	0	7	*P* = .000
<34 wk (*n*)	0	2	
Birth weight (g)	3410 [2880–4250]	2780 [980–3950]	
>10th percentile (*n*)	48	11	*P* = .020
<10th percentile (*n*)	0	11	
<5th percentile (*n*)	0	2	
